# A Latin American perspective on neurodiplomacy

**DOI:** 10.3389/fmedt.2022.1005043

**Published:** 2023-01-13

**Authors:** Mohammed A. Mostajo-Radji

**Affiliations:** ^1^ UCSC Genomics Institute, University of California Santa Cruz, Santa Cruz, CA, United States; ^2^ Live Cell Biotechnology Discovery Lab, University of California Santa Cruz, Santa Cruz, CA, United States

**Keywords:** neurodiplomacy, science diplomacy, Latin America, neurorights, neurotechnologies, neuroscience

## Overview of science diplomacy in the era of big data

Building a better understanding between nations has been crucial for the development and peace of the world. Within the field of international relations, science diplomacy has recently been in the spotlight with the mainstream media often highlighting the need for international collaborations in the development, testing and distribution of biomedical equipment, medications and vaccines related to COVID-19. Terms such as “ventilator diplomacy” and “vaccine diplomacy” have become widely used in the international arena (1, 2). Moreover, countless interactions of nonstate actors, including academic institutions and transnational youth networks have gained governmental support at unprecedent levels, particularly in Latin America (3, 4). Yet, it is important to understand that advancement of multinational interests through collaborations related to science and technology have been around us since the beginning of nations, or even before. For example, it is well established that the Silk Road served not only as a basic trade route but aided in technology development and exchange between East Asia, Persia, the Arabian Peninsula and Europe, benefiting areas such as medicine and engineering (5).

In the classic sense, multinational interests can be coordinated through special attaches that work closely with the Ministries of Foreign Affairs and other organizations. An interesting, yet understudied example is the case of the Itaipu Dam, which currently supplies 90% of Paraguay's power grid and 16% of Brazil's, making it one of the largest generators of renewable energy in the world (6). Itaipu has its roots in the Iguaçu Act of 1966, jointly signed by the Ministries of Foreign Affairs of Brazil and Paraguay, as a testimony of mutual interest to develop hydroelectric resources (6). In 1973, Paraguay and Brazil signed the Itaipu treaty to create a common strategy to exploit the hydroelectric potential of the Parana River (7). Shortly after, the company Itaipu Binacional was created to fulfill the mandate of both countries to create the dam, which was completed in 1984 (6). Since then, many binational interactions have occurred between both countries to renegotiate the terms of the treaty, which is set to expire in 2023 (8). The case of the Itaipu Dam represents a canonical form of diplomacy: direct involvement of the respective governments, appointment of special attaches, signature of treaties, coordinated development and constant revisions of agreements between parts.

Yet, diplomacy can take many forms, and it can involve many players beyond governmental agencies including: academics, the private sector, the civil society, nonprofit organizations, scientific societies, among other non-state actors (3, 9). Indeed, science diplomacy often takes the form of public and soft diplomacy: leveraging one's culture, values, resources, and policies to influence others (10, 11). Moreover, many science diplomacy efforts can be implicit, or unlabeled, making them difficult to be classified as such (12, 13). One for example, can look to large multinational collaborations, such the International Space Station (ISS) and the European Organization for Nuclear Research (CERN), which have often been highlighted as examples of science diplomacy in action due to the direct involvement of multiple governments (12, 14). In the biological sciences, the Human Genome Project (HPG) has undoubtedly been one of the most ambitious undertakes in history (15). While never formally termed a science diplomacy undertaking (16), the impact of the HGP in policy and diplomacy are immense (17): from country-wise regulations such as the Health Sector Database Act that oversees the deCODE Iceland project and the creation of a national genetic database (18), to the Universal Declaration on the Human Genome and Human Rights, led by UNESCO and adopted by the United Nations, that provides a framework to harmonizing the laws on human genomic data globally (19). Currently, lowering costs of DNA sequencing, the portability of sequencing machines such as Nanopore, and the invested interest in biodiversity and conservation, has resulted in new initiatives aiming to further develop genomics throughout the world (20). Yet, this work would have not been possible without the multinational investment and collaborations in technology development for the HPG.

The development of “big science” projects have had direct economic impact not only in the countries that were part of the initiatives but have served as a catapult for the emergence of new players in the field (21). One for example, can see how the development of several multinational astronomy observatories in northern Chile has positively impacted Chilean academia and the interest in science of local students, as well as the global perception of the country's potential in the field (22). While noteworthy, the case of Chile required large monetary investment from the local government, as well as from partners governments in the Global North, a situation that is not always possible. A different, but equally remarkable approach, has been the use of open datasets to develop local talent. To this end, African countries have made significant progress in the field of Bioinformatics (23). For instance, the H3AbioNet bioinformatics network emerged as a pan-Africa initiative to build capacity and train the next generation of African bioinformaticians through a collaboration between the African Society for Human Genetics, the US National Institutes of Health (NIH) and the UK Wellcome Trust (24). Since its beginning, the H3AbioNet has been fundamental in contributing to research, particularly in the topics of tropical diseases and HIV.

What would the next decade of science diplomacy look like? Based on current trends, I have previously speculated that a growing interest in neuroscience, combined with the generation of massive datasets, have created the need for a new field of science diplomacy: neurodiplomacy (25). Here, I will discuss the current developments in the topic, as well as the role of Latin America as a region leading several neuroscience initiatives and collaborations. For this essay, I will expand on the classic definition of science diplomacy by Van Langenhove (26), which focuses only on explicit efforts, to include implicit science diplomacy efforts which advance regional interests (25). This is done because of the lack of systematic categorization of science diplomacy efforts in Latin America (13), and to be inclusive of initiatives of interest to the Global South. In addition, this is done to include initiatives led by the civil society, such as transnational youth networks, which have been shown to play an important role in shaping policy and diplomatic efforts (10, 27).

## The need for neurodiplomacy

The fascination for the brain has long been embedded in human history. Early illustrations dating back to 300 BC Hellenic Alexandria already showed efforts to map and compare the human brain that help illustrate medical texts (28). Over 2,000 years later, we continue to explore the brain and uncover its properties through projects like the Human Connectome Project (29) and the Brain Research through Advancing Innovative Neurotechnologies (BRAIN) Initiative (30), as well as several counterparts in Europe and Asia (31). Indeed, in the last 2 decades, the number of publications of neuroscience-related articles has grown steadily every year, consistently ranking as one of the most studied fields in science (32). Remarkably, this growth has been accompanied with the appearance of new and unexpected actors, particularly those from the developing world (33). For example, Brazil and Argentina have more than doubled their yearly output of neuroscience-related articles, and now approximate the number of articles produced by European countries, such as the Netherlands and Spain (34).

Previous work has proposed “Brain-health diplomacy” as a means to mobilize transdisciplinary resources to improve brain health (35), particularly in topics related to dementia and other neurodegenerative disorders (36). However, I argue that brain health diplomacy is not inclusive enough of all the emerging issues and areas related to the human brain, including: human rights, the generation and ownership of large datasets to map brain wiring and function, and education initiatives in neuroscience. Neurodiplomacy on the other hand, is inclusive of these and other topics (25), as will be discussed below.

In 2017, neurorights were proposed as a needed advancement in basic human rights in the era of neuroscience (37). Specifically, 4 areas were identified as relevant frameworks for future ethical studies and legislation: cognitive liberty, mental privacy, mental integrity, and psychological continuity (37). The authors argued that these rights fulfilled the criteria for human rights, as defined by Philip Alston (38). Namely, these rights are fundamentally valuable to society, are consistent with international human rights law, are precise enough to give rise to rights and obligations, and are likely to achieve international consensus (37, 38):

1) **Cognitive liberty:** The concept of cognitive liberty expands on traditional definitions of freedom of thought by also excluding the possibility of an individual being coerced to a thought by neurotechnologies (39). In other words, cognitive liberty allows the individuals to refuse coercive uses of neurotechnologies (37). In complement, the right to cognitive liberty, should, in principle, include positive formulations, such as equal access to the neurotechnologies themselves, if those technologies are deemed ethical (39). Given the close relation between cognitive liberty and the universal principles of freedom, it is likely that these rights will be widely accepted by the global community.
2) **Mental privacy:** The right to privacy is recognized by several international conventions, including the Universal Declaration of Human Rights and the European Convention on Human Rights (37). However, these conventions have fallen behind at addressing the right of privacy of the mind and thoughts (40). This is particularly problematic because the data generated can be used to directly track the source of the data (40). For example, brain waves and patterns can be used as biometric identifiers (41), although they can be obtained without an individual being aware of the collection of such data (37). Moreover, the use of neuroimaging technologies and other recordings on brain waves in criminal investigations could violate the privileges against self-incrimination, recognized by most legal proceedings in democratic countries, as well as international conventions (42, 43). Therefore, new rights that specifically address the unique challenges of brain data need to be discussed and agreed on in the global arena.
3) **Mental integrity:** This right extends on mental privacy, by also protecting individuals from intrusion that could alter neuronal activity and computation to cause self-harm (37, 44). While many states recognize the right to body integrity, it can be argued that the right to mental integrity remains unclear in current legislations (45). In other words, while it is clear to states that nonconsensual interference with one’s body is forbidden, interfering with once’s mind, such as deliberately causing mental suffering, is not necessarily covered by national or international law (45). Therefore, and considering newer technology developments, further discussions are needed to fill this normative gap.
4) **Psychological continuity:** Several changes to the mind can be done without violating mental privacy and integrity, such as modification of emotions, impulse control, and induction of pain (37). Violations to psychological continuity can sometimes be subtle and undetected by the individuals, such as the use of marketing technique to control one’s behavior and preferences (46). It is therefore understandable that the right to psychological continuity is not currently regulated (47).

Simultaneously to the proposal of neurorights, 4 areas of concern in neurotechnologies and artificial intelligence were identified by neuroscientists and ethicists as priorities to be addressed: privacy and consent, agency and identity, augmentation, and bias (48).

1) **Privacy and consent:** Data trails can be used to infer a great variety of personal information, including demographics, behavior, and personality traits (49). For example, mobility patterns tracked during the COVID-19 pandemic have been used to extrapolate personality traits that could be considered risky behaviors for viral transmission (50). With the rise of Internet-enabled neurotechnologies (51) mechanisms that safeguard user data and privacy are needed to ensure the protection of neurorights.
2) **Agency and identity:** Given that the majority of neurorights are concerned with protecting individuals’ mental integrity and psychological continuity, new normative frameworks need to be discusessed at the international level to create conventions that guarantee respect to the individual’s self and sense of personal responsibility (48).
3) **Augmentation:** If available, technologies and drugs that enhance mental abilities are likely to be widely adopted (48). For example, college campuses have already experienced an abuse of neurostimulants such as Adderall and Ritalin, which are perceived as enhancing cognitive abilities (52). Moreover, several countries have invested in cognitive enhancement for military purposes (53), which to date remains underregulated. Given the advantages that cognitive augmentation can provide, normative frameworks that guarantee an equal and culturally sensitive access to these technologies will be needed.
4) **Bias:** In the artificial intelligence field, there are several clear examples of algorithm bias toward specific genders and racial identities, which emerge, at least in part, by the training datasets (54). In genomics, the reference human genome is of male white European background, which potentially leaves behind other populations in several risk allele studies (55). As technologies to understand and heal the brain emerge, it is important to reduce potential biases that can unfairly target underrepresented groups.

From its origins, it was understood that neurorights would intrinsically be linked to diplomacy, as several of the normative agreements, such as the Declaration of Human Rights, depended on multinational organizations, including the United Nations (48). Yet, the link between neuroscience and diplomacy encompasses areas beyond human rights. With a combined global market size of over $200 billion, neurotechnologies and the treatment of neurological diseases should be seen as an important area of international trade. Therefore, I have proposed neurodiplomacy as a needed field to encompass multinational efforts and regulations related to the brain and nervous system from collaborative scientific endeavors between multiple countries and governments, to international trade and human rights (25).

## Neuroscience initiatives in Latin America are setting the ground for neurodiplomacy

**Figure 1:**
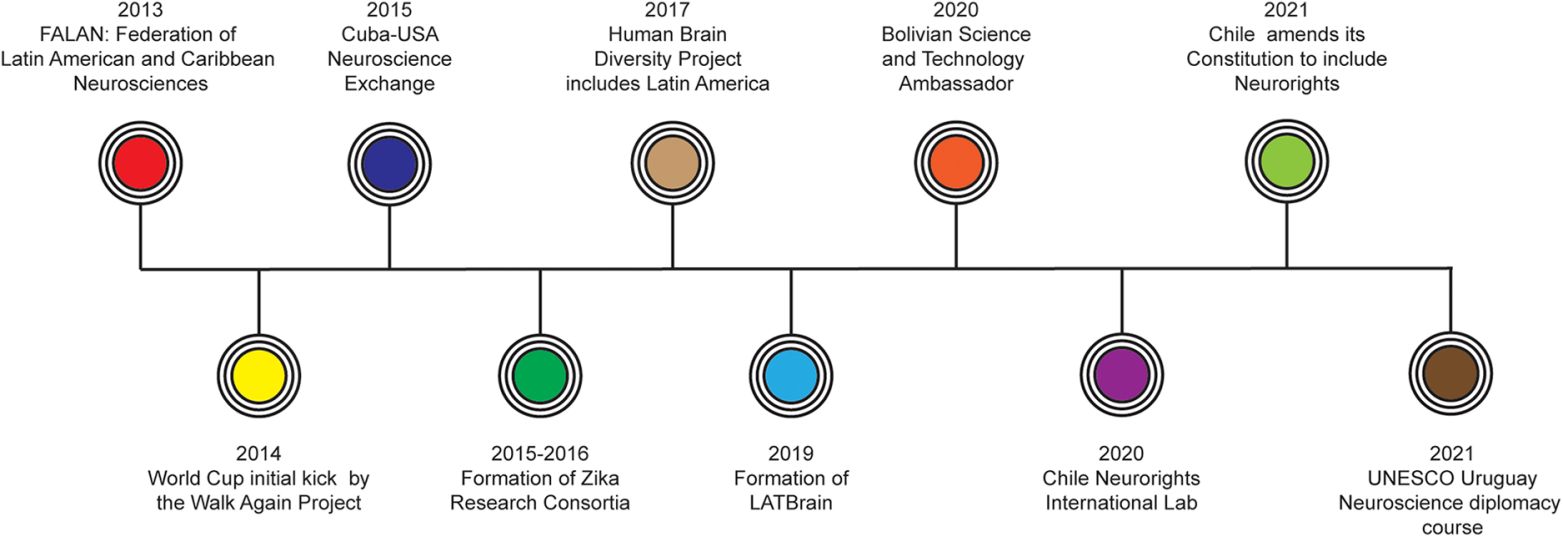
Major landmarks of neurodiplomacy and neuroscience initiatives in Latin America.

While important neuroscience-based initiatives are happening throughout the world (56), I will focus on the case of Latin America as the region has started to gain momentum as a leader in neurodiplomacy (Figure 1). Several of these initiatives have had direct involvement of governments either through multinational agreements or targeted funding. Others have involved non-state actors such as universities, research institutes and nonprofit organizations (3, 27). Moreover, many initiatives can be considered people-to-people diplomacy which have the potential to pave the way to policy changes and multinational collaborations (10).

An early example of a multinational neuroscience-based collaborations is the Walk Again Project (WAP) led by the Brazilian neuroscientist Miguel Nicolelis. As result of a collaboration of more than 150 scientists and engineers from around the world, Brazil made history by having a paraplegic man aided by a robotic exoskeleton give the initial kick in the 2014 World Cup opening ceremony in Sao Paulo (57). This project, which was funded for multiple years by the United States and the Brazilian governments, among other entities, has had lasting effects in the Brazilian education system. For instance, the Santos Dumont Institute has become a premier institution in Brazilian Northeast that is currently driving neuroscience and bioengineering education and research, and a steppingstone toward the “Knowledge Island” proposed earlier by Nicolelis, Sidarta Ribeiro and Claudio Mello (58). It can be argued that having the largest Latin American country highlight the power of neuroscience at a global event set the tone for the continent. For example, the Federation of Neuroscience Societies in Latin America, the Caribbean, and the Iberian Peninsula (FALAN) was created in 2013 to promote neuroscience research and knowledge exchange in the region. That same year, the Grass Foundation awarded the Society for Neuroscience over half a million dollars to create a Latin American Neuroscience Training Program (59). It should therefore come to no surprise that for the first time the International Brain Research Organization (IBRO) hosted its World Congress in Latin America in 2015, picking Brazil as the host country.

At the diplomatic level, several approaches have been taken by Latin American countries to introduce science, technology, and innovation development into their governmental spheres (9, 13). Panama, for example, was the first Latin American country to formally incorporate science and technology as instruments of their foreign affairs agenda in 2018 (9, 13, 60, 61). Other countries, like Ecuador, Uruguay, Colombia, and Mexico have incorporated attaches within their existing diplomatic missions that oversee specific collaborative projects (9, 13). In 2014, Costa Rica took a different approach and named a scientist as its ambassador to the United States to push forward a collaborative agenda between both countries. In 2020, Bolivia pioneered neurodiplomacy by becoming the first developing country to name a neuroscientist as a global ambassador for Science, Technology and Innovation (9, 13, 62). It is important to mention that while Latin America has frequently sought collaborations with the United States, the United States has also reciprocated the efforts. For instance, upon thawing of the diplomatic tensions between the United States and Cuba, a delegation of scientists from the United States visited Cuba as part of a program sponsored by the American Association for the Advancement of Science (AAAS), where they met their Cuban counterparts and identified 3 areas of neuroscience to start scientific collaborations and personnel exchange: neuroimaging and neuroinformatics, neurodevelopment and nonhuman primate neuroscience (63). Similarly, the United States Department of State has sponsored several exchange programs with Latin America. A prominent example has been Clubes de Ciencia (Science Clubs), that has allowed hundreds of US-based scientists to collaborate with their Latin American counterparts and train thousands of Latin American high school and college students (33, 64).

Finally, non-state actors, including academics, nonprofit organizations and entrepreneurs have also played a major role in advancing neuroscience collaborations toward the Sustainable Development Goals (3, 62). IBRO and FALAN, for example, have sponsored countless conferences and exchanges within the region, as well as with organizations outside Latin America. The governmental-sponsored business incubator Start-Up Chile has aided low-cost neuroscience teaching equipment companies, such as Backyard Brains, to penetrate the Latin American market. In the academic sector, the major driver of capacity building in the neurosciences was the Zika outbreak of 2015–2016. During this outbreak, several consortia were formed within Latin America, as well as partnerships with the Global North. In 2016, the European Commission awarded 3 grants for the formation of intercontinental consortia researching Zika to ZikaPLAN, ZIKAction and ZIKAlliance (65). Through these and other consortia, Latin America has positioned itself as a key player in Zika research, collaborating with partners in the United States, Canada, Europe, Asia, Oceania and Africa. At the same time, the region has used this opportunity for capacity building. For example, the Neuroviruses Emerging in the Americas Study (NEAS) set up at least 10 clinical sites in Colombia, which has served as major clinical sampling sites (66). This capacity building in return, has resulted in higher interest in including the region in global efforts. For instance, the Human Brain Diversity project, an effort to shed light into the variability of brain physiology throughout the world using electroencephalogram recordings, included Latin America in 2017 (67).

## The future of neurodiplomacy in Latin America

With the lowering cost of data generation and growing interest in big data projects that include diverse samples, we are in the midst of a revolution in neuroscience. It is therefore our duty to include all actors, both governmental and non-state actors, in the decision-making process. I proposed the coining of the term “Neurodiplomacy” to encompass science diplomacy focused on neuroscience (25). I further suggest that at least six areas of interest require our immediate attention: Neurorights, infectious diseases, data governance, trade of neurotechnologies, education and people-to-people exchanges. While each of them can be the topic of its own review, I will briefly describe the need of each of them:

1) **Neurorights:** As described above, given that current human rights are limited in their scope and do not encompass the special situations generated by the development of neurotechnologies, high-level discussions are needed to protect the privacy and integrity of humanity (37). It is important to mention that because the Universal Declaration of Human Rights is not legally binding (68), the conversations will be needed in multiple arenas and involving multiple states and non-state actors. For example, the European Convention on Human Rights is a multinational, legally binding treaty that is overseen by an international court (69). While not exactly a counterpart, the American Convention on Human Rights is the treaty led by the Organization for American States (OAS) and ratified by most Latin American countries (70). However, both the convention and the OAS have been strongly criticized leading to the withdrawal of two countries, namely Trinidad and Tobago (71) and Venezuela (72). Given that Chile has been a pioneer in integrating neurorights into law, it is likely that it will lead several of these conversations (73). But while it is agreed on that mental privacy, integrity and continuity should be morally protected, whether specific neurorights are needed is still up to debate (74). Indeed, several scholars argue that the adoption of neurorights would lead to unnecessary rights inflation (74, 75). It is only through open discussions and debate that an agreement can be reached.
2) **Infectious diseases:** Despite significant investments by the World Health Organization and local governments, infectious diseases remain among the top causes of death throughout the world, particularly in low- and middle-income countries (76). Moreover, the effective fight against infectious diseases usually requires transnational coordination and funding (77). In Latin America, the outbreak of Zika has been a seen as a primary example of science diplomacy in action, which required the coordinated involvement of multiple governments, multinational organizations and non-state actors, such as universities and research centers (77). Among the long-lasting effects of COVID-19 are damages to the brain and the peripheral nervous system (78, 79). Importantly, the molecular mechanisms of SARS-CoV-2 entry into the brain seems to be different than to other organs (80). Specifically, ACE2, the primary gene for SARS-CoV-2 entry to the lungs, is not expressed in brain cells (80), and SARS-CoV-2 has been shown to infect astrocytes in brain tissue through, at least in part, DPP4 and CD147 (80). Therefore, new collaborations that tackle COVID-19 as a brain infectious disease may be needed to effectively target these affections. In addition to COVID-19 and Zika, neuropathologies are observed in 70%–90% of AIDS patients (81). This is of relevance to Latin America, as following the COVID-19 pandemic there has been a peak in HIV infections in low- and middle-income countries due to unregulated convalescent plasma transfusions (82).
3) **Data governance:** Currently, there is a lack of coordination throughout the world in relation to data governance (83). This situation effectively leaves the compliance responsibility and liability to individual investigators and labs, which hampers discovery (84). Neuroscience has an increasing need for datasets that are larger than what a single laboratory could obtain (83). Moreover, data generated through global or regional initiatives are subject to different privacy rules, as defined by the geographical borders in which the data was obtained or by specific mandates of the funding agencies (83). Because countries have different levels of stringency when it comes to privacy regulations and laws, and because neuroscience data can be directly used to identify individuals (40), reaching an international agreement on data governance and sharing is needed. Contact tracing software developed during the COVID-19 pandemic unveiled that in relation to the rest of the world, Latin American countries have weak regulations regarding data governance (85). Therefore, initiatives that better educate policy makers, scientists and the public in this region are imperative (83).
4) **Trade of neurotechnologies:** In the global market, there is a growing momentum for the development of neurotechnologies for applications in both research and treatments of neurological disorders (86). Recent advances have significantly lowered the price of neurotechnologies, allowing their use in the developing world (87, 88). However, these technologies will need to undergo stringent regulatory approvals to be deployed to the masses (89). Latin America has peculiarities in its geography that have influenced their population (90). Indeed, these adaptations should be considered in biomedical technology design (90). For example, South America is home to the larger population of people living in high altitude (90). In the brain, several anatomical structural changes have been shaped by adaptation to high altitude (91), including reduction of the grey area in the insula, lingual cortex, and the prefrontal cortex (92), as well as changes in the white area (92). In addition, adaptation to hypoxia conditions experienced in high altitude include a hypometabolism in the brain (93), and structural changes in the blood-brain barrier (94). The relationship between these anatomical changes and the effectiveness of neurotechnologies still remain an open question, but given the projected growth and integration with society of these technologies (95), it should be a focus of interest to the region.
5) **Education:** Education has long been recognized as a powerful tool for soft diplomacy (33, 96, 97). Yet, neuroscience research and education remains highly unequal in Latin America. Brazil, for example, leads neuroscience production in the continent (98–100). Other countries with relatively high neuroscience publications are Mexico, Chile, Colombia, and Argentina, while the numbers of neuroscience articles from other countries is small (99, 100). Several reasons can be attributed to this phenomenon, including political stability, economic prosperity, and cultural norms (101). Even within individual Latin American countries there are biases that leave behind women, sexual minorities, and underrepresented groups (101). Therefore, creating regional policies that can overcome these barriers are needed to equalize the field within Latin America, while allowing the region to catapult itself globally.
6) **People-to-people exchanges:** Unlike other forms of public diplomacy, people-to-people exchanges include a “human” factor, in which the development and psychology of the participants are key elements of the intervention (102). For example, the Fulbright program, sponsored by the United States Department of State, has been a key soft diplomacy tool to advance the interests of the United States throughout the world (103). Since the 1950s, the Fulbright program has allowed thousands of Latin American students and professionals to received training in the United States, while at the same time encouraging US citizens to train in Latin America (104). Several Latin American countries lead similar exchange initiatives. In Brazil, for instance, the Ciência sem Fronteiras (Science without Borders) program has allowed for the internationalization of Brazilian universities, while creating positive outcomes in STEM (105). The effects of mobility in scientific collaborations are strong, as countries with higher mobility rates are often benefiting from publishing with scientific partners in more prolific countries (106). In order to strengthen the emerging role of Latin America in neuroscience, more exchange programs that not only target mobility with the developed world, but also promote regional exchanges will be needed (107).

I used the example of Latin America as a new player in the field of neurodiplomacy. While countless multinational collaborations are in place throughout the world, the case of Latin America is remarkable, as the exchange between governments and neuroscientists has been steadily increasing over the past decade. In 2019, for example, LATBrain was created, as a multinational multi-institutional agreement to promote neuroscience research and neurotechnology-based economies in the region (108). In 2020, the Neurorights International Lab was established in Chile, and shortly after, Chile became the first country in the world to approve a constitutional amendment to include neurorights as human rights (109). The interactions with academia have not only been driven by the home governments but also by multinational organizations. For example, the United Nations Educational, Scientific and Cultural Organization (UNESCO) recently sponsored a science diplomacy focused on neuroscience workshop in Uruguay (110). To my knowledge, this is the first neuroscience-specific science diplomacy workshop in the world and a sign that neurodiplomacy in the region will only keep increasing.

What would the future of neuroscience collaborations look like? Many opinions exist on how scientific discovery and education will continue in the post COVID-19 era. It is true that Latin American countries spend, on average, less than 1% of their GDP on research (101). Therefore, approaches that are permissible to low-cost scalability will likely be prioritized. While people-to-people exchanges will likely return, new approaches have also been suggested. For example, low-cost Internet-of-Things (IoT)-enabled equipment manipulation and data acquisition can become an integral part of laboratory practices (25, 111, 112). If adopted by the community, this approach could enable open data generation and accessibility transcending borders, allowing new avenues for global collaborations in research and education, as it has been previously seen in the field of genomics. Indeed, this approach of next generation “brain observatories” or “IoT-enabled shared labs” has been recently proposed as a mean to democratize access to neuroscience research (113), and have already been tested in the context of neuroscience education in the United States and Latin America (111).

## Conclusion

I proposed the term Neurodiplomacy to encompass the study and the actions that can be undertaken at the intersection of neuroscience and international policy (25). In this article, I used the example of Latin America to illustrate some of the initiatives, advances and issues that are common to the region. I further proposed that given its strength and weaknesses, Latin America should pay special attention to 6 areas of neurodiplomacy, namely: neurorights, infectious diseases, data governance, trade of neurotechnologies, education and people-to-people exchanges. Given the broad spectrum of these subfields, one may wonder which of those should be prioritize. Yet, it is important to remember that those areas are intertwined. For example, education and people-to-people exchanges can be used as capacity building tools to create common strategies to address infectious diseases. Similarly, and as discussed above, neurorights are proposed to, among other things, safeguard people from misuse of neurotechnologies. Correspondingly, many of the issues with neurotechnologies are related to data governance and sharing. It is therefore expected that, as seen with other development goals, the advancement of one goal will move forward the rest (3).

It is also important to point out that the areas selected for discussion in this article are based on my experience as a Latin American neuroscientist and former diplomat. It is my belief that the proposed areas of advancement address regional issues, while taking advantage of Latin America's unique cultural diversity and wealth. A similar analysis of other regions is needed to further amplify neurodiplomatic efforts throughout the world. Ultimately, this article is meant to open a debate and position Latin America at the forefront of neurodiplomacy.
